# Evaluating Population Receptive Field Estimation Frameworks in Terms of Robustness and Reproducibility

**DOI:** 10.1371/journal.pone.0114054

**Published:** 2014-12-02

**Authors:** Mario Senden, Joel Reithler, Sven Gijsen, Rainer Goebel

**Affiliations:** 1 Department of Cognitive Neuroscience, Faculty of Psychology and Neuroscience, Maastricht University, P.O. Box 616, 6200 MD Maastricht, The Netherlands; 2 Maastricht Brain Imaging Centre, Faculty of Psychology and Neuroscience, Maastricht University, P.O. Box 616, 6200 MD Maastricht, The Netherlands; 3 Department of Neuroimaging and Neuromodeling, Netherlands Institute for Neuroscience, an Institute of the Royal Netherlands Academy of Arts and Sciences (KNAW), 1105BA Amsterdam, The Netherlands; Harvard Medical School/Massachusetts General Hospital, United States of America

## Abstract

Within vision research retinotopic mapping and the more general receptive field estimation approach constitute not only an active field of research in itself but also underlie a plethora of interesting applications. This necessitates not only good estimation of population receptive fields (pRFs) but also that these receptive fields are consistent across time rather than dynamically changing. It is therefore of interest to maximize the accuracy with which population receptive fields can be estimated in a functional magnetic resonance imaging (fMRI) setting. This, in turn, requires an adequate estimation framework providing the data for population receptive field mapping. More specifically, adequate decisions with regard to stimulus choice and mode of presentation need to be made. Additionally, it needs to be evaluated whether the stimulation protocol should entail mean luminance periods and whether it is advantageous to average the blood oxygenation level dependent (BOLD) signal across stimulus cycles or not. By systematically studying the effects of these decisions on pRF estimates in an empirical as well as simulation setting we come to the conclusion that a bar stimulus presented at random positions and interspersed with mean luminance periods is generally most favorable. Finally, using this optimal estimation framework we furthermore tested the assumption of temporal consistency of population receptive fields. We show that the estimation of pRFs from two temporally separated sessions leads to highly similar pRF parameters.

## Introduction

An essential aspect of vision research using functional magnetic resonance imaging (fMRI) is the investigation of retinotopic organization of visual cortex [1], [2]. Phase encoded retinotopic mapping as pioneered by Sereno et al. [3] already allowed for the systematic investigation of polar angle and eccentricity properties of visual cortex. Recently, the advent of the population receptive field (pRF) mapping approach, first described by Dumoulin and Wandell [1], has supplemented knowledge of receptive field location with insight regarding their size and shape. Beyond the immediate scientific interest in receptive field properties, knowledge of receptive fields is crucial for a number of applications. Receptive fields a) provide a source of information for the reconstruction of stimuli from the blood oxygenation level dependent (BOLD) signal [4], b) can serve as target for transcranial magnetic stimulation [5], c) assist function based alignment, d) provide a spatial forward model for computational models [6], and e) might give important insights with respect to theories of attention [7] as well as into pathologies of visual cortex [8]–[10] and brain development.

For the utilization of receptive fields for any of the aforementioned applications, it is necessary that they are measured, or rather estimated, with a high degree of precision. For a number of applications it is necessary to estimate pRF parameters from one set of stimuli and use their predictions on a distinct set of stimuli. For other applications it is necessary to perform estimation in one session to be able to use the obtained parameters in future sessions. For these reasons, the precision of pRF estimation necessarily pertains to generalizability across stimuli as well as across sessions (i.e. time).

**Figure 1:**
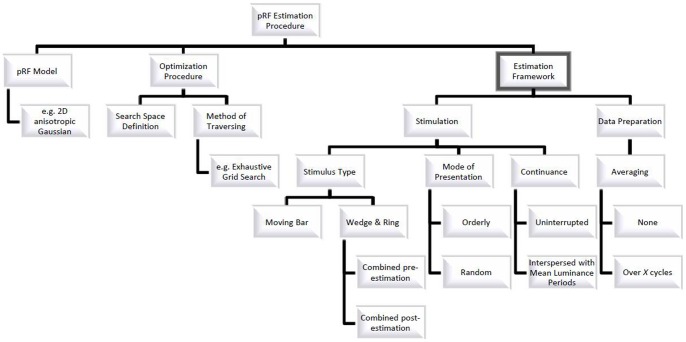
Conceptual Division of the pRF Estimation Procedure. This figure gives a visual organization of the pRF Estimation Procedure with its components: 1) pRF Mode, 2) Optimization Procedure, and 3) Estimation Framework. The frame around Estimation Framework indicates that choices related to this component and its subcomponents constitute the focus of this paper.

Such precision relies first and foremost on three aspects of the estimation procedure (see figure 1 for a visual representation of this organization). First of all, an adequate model description of a receptive field is needed to capture its position, shape, and local properties. Secondly, it is necessary to define an accurate and fast optimization procedure by specifying both a suitable search space on the parameters and an efficient way to traverse this space. Finally, it is necessary to setup an adequate estimation framework providing the data from which receptive fields are estimated. With regard to model description and optimization procedure other groups have previously produced excellent work [1], [2], [11]. According to our knowledge, however, the estimation framework has so far not undergone a thorough investigation and it is our aim to provide a first attempt by studying how different choices affect pRF estimation performance.

In detail, the estimation framework encompasses stimulation and data preparation. Stimulation, in turn, refers to the type of stimulus used (e.g. moving bar), stimulus presentation (the stimulus changes location in an orderly or random fashion), and continuance (stimulation is continuous or interspersed with mean luminance periods). Importantly, the stimulus type does not necessarily refer to a single stimulus but can also refer to an integration of at least two stimuli. For instance, stimuli which can only reveal partial information with respect to receptive field location (rotating wedge and contracting ring only convey information on polar angle and eccentricity, respectively) can be integrated into a single stimulus (Wedge-Ring or WR). This integration of information is performed independent of stimulus presentation. In the case of wedge and ring stimuli each stimulus is presented individually and only after acquisition of the BOLD signal for each is the information integrated. This, however, can be done before or after estimation of pRF parameters. Specifically, integrating the information before estimation is at the level of the signal. It involves the concatenation of signals originating from runs presenting each of these stimuli individually. Estimation is then performed on the concatenated signal. Integrating the information after estimation is at the level of the parameters. In this case, parameters are first estimated separately for each stimulus and the relevant parameter estimates from each stimulus are retained and combined to generate a full set. Here we considered three stimulus types: bar, wedge-ring pre-estimation (WR_pre_), and wedge-ring post-estimation (WR_post_) and supplement the more commonly used orderly presentation of stimuli with random presentation. We include a random presentation sequence since it had previously been shown that a pseudo-random, multi-focal, stimulus improved pRF estimation in the presence of foveal scotomas [9]. It is as of yet unclear, however, whether this improvement was due to the multi-focal nature of the stimulus or it being presented in a pseudo-random fashion and whether such choices have any benefit in healthy vision. Regarding continuance, we systematically investigated the effects of including mean luminance periods since it has previously been suggested that their inclusion provides a baseline that allows for improved estimation of large receptive field sizes higher up in the visual hierarchy [1]. Lastly, data preparation refers to averaging the BOLD signal across stimulus cycles in order to average out random noise in the signal. While averaging should increase the proportion of variance in the BOLD signal that is explained by predictions generated from pRF parameters, it is not clear whether higher proportions of explained variance reflect an improvement in pRF estimation or are simply due to a reduction in the total amount of variance to be explained. We aim to distinguish between these possibilities in our evaluation of the effects of averaging.

While we hope to improve the estimation of pRFs and hence their utility to other aspects of vision research, it is apparent that although good estimation of pRFs is surely necessary, it is not always sufficient. That is, it is also necessary to assume that pRFs are stable across time in the healthy adult brain a) for situations in which functional localizers and experimental fMRI data are acquired in separate sessions, b) for function based alignment, c) to provide a spatial forward model for computational models, and d) for studying changes in the diseases affecting retinotopic organization [8] and the developing brain. Therefore, the second aim of our paper is to test this assumption of stability or temporal consistency.

In order to achieve these aims we study how different choices affect pRF estimation performance in both an empirical as well as in a simulation setting. Simulations allow us to have knowledge of the ground truth regarding pRF size and shape. This renders it possible to investigate in how far choices related to the estimation framework produce correct estimates of each pRF parameter rather than simply explaining variance within the measured BOLD signal.

In summary, we aim to 1) provide a first guideline with respect to choices relating to the estimation framework, and 2) establish the temporal consistency of receptive fields in the healthy adult brain in order to provide a good rationale for vision research relying on knowledge of population receptive fields as well as to further our understanding of them.

Our results with regard to the estimation framework show that a bar stimulus presented in a random (non-continuous) fashion produces the most promising estimates of pRFs, especially if stimulation is interspersed with mean luminance periods. Furthermore, averaging of the BOLD signal reduces the amount of variance left unexplained in the signal and might thus convey an advantage in delineating visually responsive voxels. Additionally, for very noisy (high resolution) data sets, our simulations suggest that heavy averaging leads to more accurate pRF parameter estimates while it does not do so if the noise is moderate. Our results with regard to temporal consistency show that pRF estimates originating from two separate measurement sessions separated by a week appear to be very consistent especially with regard to their location and size. The angle of elongation of anisotropic receptive fields, however, is subject to moderate variations as our simulation results revealed that it is generally more difficult to estimate than the other parameters.

## Materials and Methods

### Participants

Two fMRI measurements separated by 1 week were obtained from three male volunteers. All volunteers were without prior history of psychiatric or neurological illness. The ages in years of subjects one, two, and three were 34, 27, and 35, respectively. All subjects were right handed and had normal or corrected-to-normal visual acuity, were screened, and provided written informed consent prior to scanning.

### Ethics Statement

The Ethics Committee of the Faculty of Psychology and Neuroscience at Maastricht University approved this study and the procedures employed therein.

### Stimulus Description

**Figure 2:**
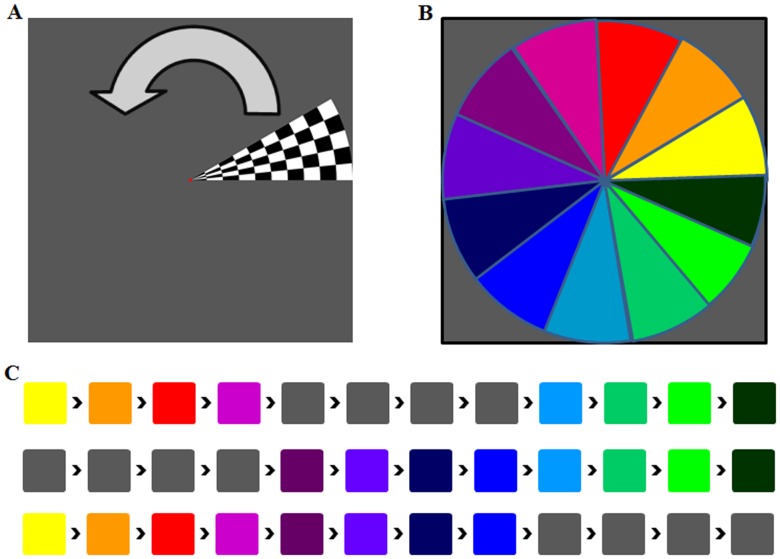
Schematic of Stimulus Presentation Scheme. This figure shows a representative schematic of the stimulus presentation scheme including mean luminance periods using the orderly presented wedge stimulus as an example. **A)** Shows a standard wedge aperture located at 0° revealing a checkerboard pattern. The wedge turns counter-clockwise. **B)** Shows a color-code for each of the twelve wedge positions presented during stimulation. **C)** Shows the timecourse of stimulus presentation for three exemplary cycles. The entire stimulation consists of 15 such cycles. The colors of the squares correspond to the color of the wedge position shown at a certain moment in time lasting for two seconds ( = 1TR). Grey squares indicate mean luminance periods.

In the present study we used the conventional wedge, ring, and bar stimuli composed of a high-contrast, moving, checkerboard pattern [1]–[3], [12], [13]. In contrast to common practice the stimuli did not move continuously across the visual field but in 12 discrete steps and remained at each position for 2 seconds ( = 1 TR), covering the entire visual field within 24 seconds. The wedge therefore subtended 30° (1/12^th^ of the entire 360°) and the width of the rings and bars was 1/12^th^ of the maximum stimulus radius and diameter, respectively. We presented all stimuli in two different modes. The first mode was consecutive with each stimulus position bordering the previous position. For the wedge and ring stimuli this coincides with the phase encoded design first described by Sereno et al. [3]. In the first mode the wedge was presented counter clockwise while the ring was contracting and the bar was presented in four different orientations (0,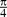, 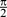, and 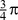 radians) moving in two different directions for each of the orientations (orthogonal to orientation). In the second mode the stimuli were shown at random positions with the exception that for the bar stimulus the positions were only randomized within an orientation and never across orientations. Similar to the original pRF estimation procedure described by Dumoulin and Wandell [1] we inserted mean luminance periods during which the participants only saw a zero contrast field. Within each cycle mean luminance fields replaced the stimuli for four consecutive TRs. This amounts to a total of 8 seconds within each cycle wherein subjects were shown zero contrast rather than a stimulus. The order at which mean luminance fields were inserted was pseudorandom; that is, for each cycle four different positions were replaced such that each position was presented equally often. Figure 2 shows a schematic of mean luminance insertion into stimulus cycles using the orderly presented wedge stimulus as an example. For both wedge and ring 15 cycles were presented. For the bar each combination of orientation and direction (4*2) consisted of one cycle. There were thus 8 cycles to show each of these combinations and each combination was shown 3 times resulting in a total of 24 cycles. Illustrative visualizations of all stimulus presentations for subject one are provided in the form of compressed video files in File S1 (all videos are played at fourfold speed of actual stimulation and checkerboards are static rather than flickering).

### Stimulus Presentations

The open source stimulus presentation tool StimulGL, developed by authors SG and RG (https://sites.google.com/site/stimulgl/) was used for presenting the various visual stimuli to the subjects at a screen resolution of 1680×1050 pixels. The stimuli had a resolution of 1050×1050 pixels. The experiments were performed on a hardware configuration containing a Dell Optiplex 970 computer with a NVIDIA NVS 300 graphics card with OpenGL >2.0 support connected to a Panasonic PT EZ570E wuxga projector. The projected visual stimuli were reflected first with a mirror behind the bore of the magnet and secondly by a mirror above the head coil to the subject. The stimuli filled an area of 40 (width) x 23.5 (height) cm^2^ when projected onto the scanner’s frosted screen, corresponding to 30×18 degrees visual angle. Given the limit of 18° visual angle in the vertical direction, all stimuli were also limited to 18° in their horizontal extent.

### Magnetic resonance imaging

Imaging data were acquired using a 3T Tim Trio scanner equipped with a 32-channel head coil (Siemens Medical Systems, Erlangen, Germany). Anatomical data were collected with a T1-weighted MPRAGE imaging sequence (192 sagittal slices; Repetition Time [TR] = 2250 ms; Echo Time [TE] = 2.17 ms; Flip Angle [FA] = 9°; Field of View [FoV] = 256×256 mm^2^; 1 mm isotropic resolution; GRAPPA = 2). Functional images were acquired using a gradient-echo echo-planar imaging sequence (31 transversal slices; TR = 2000 ms; TE = 30 ms; FA = 77°; FoV = 216×216 mm^2^; 2 mm isotropic resolution; no slice gap; GRAPPA = 2).

### Processing of (f)MRI data

All imaging data were analyzed using BrainVoyager QX (v2.6; Brain Innovation, Maastricht, the Netherlands). Anatomical datasets underwent brain extraction, followed by inhomogeneity correction and transformation to ACPC space. Preprocessing of the functional datasets followed standard procedures including slice scan time correction, (rigid body) motion correction, linear trend removal, and temporal high-pass filtering (up to 2 cycles per run). Head motion was minimal (<1.5 mm translation and <1.5° rotation in any direction in all runs of every subject) and corrected successfully during post-processing. Due to the use of preparation scans, none of the initial volumes needed to be discarded related to T1 equilibrium effects.

### Model Based Analysis

We loosely followed the procedure described by Dumoulin and Wandell [1] for estimating the pRF parameters from the time series data using a linear spatio-temporal model of the fMRI response. Specifically, after the BOLD signal measured for each voxel is z-normalized, it is modeled by
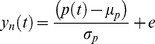
(1)where *p*(*t*) is the predicted BOLD signal, *e* represents additive Gaussian noise, and µ_p_ and σ_p_ respectively represent the mean and standard deviation of the predicted BOLD signal. In accordance with Dumoulin and Wandell [1] the prediction *p*(*t*) was calculated from a parameterized model of the underlying modeled neuronal population receptive field and the stimulus. We estimated the pRF model by finding those model parameters that best fit the data. Here, we modeled the pRF as an anisotropic two-dimensional Gaussian. We used an anisotropic model for reasons of completeness as it allows us to investigate the aptitude of each stimulus in identifying all pRF properties. Additionally, previous research had shown that an anisotropic model is beneficial for the estimation of pRF location at eccentricities close to the outer boundaries of the stimulated visual field segment [2]. We did, however, repeat the analyses with an isotropic pRF model and get the same results with regard to stimulus choice. The anisotropic Gaussian is defined by five parameters (x_0_,y_0_,σ_x_,σ_y_,θ),
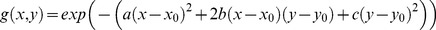
(2)and



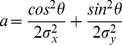
(3)

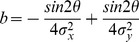
(4)




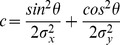
(5)where (x_0_,y_0_) is the center of the Gaussian, σ_x_ and σ_y_ is the spread in the X and Y directions, respectively, and θ is the rotation of the Gaussian. From this receptive field model and an effective stimulus, *s*(*x*,*y*,*t*), which is a binary indicator function that marks the position of the stimulus aperture at each time, we calculated the BOLD signal in two steps. First, we calculated the overlap between the effective stimulus and the model pRF at each point in time:



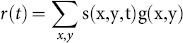
(6)


Second, we convolved this overlap with a two-gamma hemodynamic response function *h*:
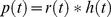
(7)


In order to find the optimal pRF parameters we performed a grid search. The grid consisted of a set of 36000 plausible parameter configurations. Specifically, we used a 20 by 20 grid of central positions ranging from −9° to +9° (measured from fixation), a set of 10 exponentially increasing spreads from 0.25 to 10 (we expected most RFs to be comparatively small and only a few to be big), two ratios of spread in the X and Y direction (σ_x_/σ_y_ = 1∶1 and 2∶1), one angle of rotation if the ratio is 1∶1, and eight angles of rotation if the ratio is 2∶1. The search space was traversed for all voxels simultaneously using a graphical processing unit (GPU) accelerated parallelization procedure. Each voxel was assigned the parameter set which minimizes the normalized root-mean-square error (NRMSE) between its Z-normalized predicted and Z-normalized measured BOLD signal:
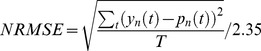
(8)where T is the number of time points, 2.35 is the full width at half maximum of the Z-distribution, and the subscript *n* indicates that the data is normalized to Z-scores. Additionally, a fit value was retained for each voxel given by 1-*NRMSE*.

### Information Integration

With respect to pRF location the wedge and ring stimuli can arguably only provide adequate information on either polar angle or eccentricity, respectively. We, therefore, only considered combinations of these stimuli in estimating pRF parameters. Specifically, we considered two modes of combination. For the first mode the stimuli were combined before pRF estimation by concatenation of the BOLD signals obtained for each stimulus individually. The pRF parameters were then estimated from this concatenated signal (we refer to this stimulus setting as WR_pre_). For the second mode the pRF parameters were first estimated for each stimulus individually. Afterwards, the Cartesian coordinates obtained for the wedge were transformed to angular values while those of the ring were transformed to radii. Next, the combined location was obtained by transforming these angle and radius values back to the Cartesian coordinate system. Finally, the remaining parameter values were averaged across the two stimuli (we refer to this stimulus setting as WR_post_). To obtain the average of the rotation angle we used the formula for the mean of circular quantities. The bar stimulus (hereafter referred to as bar) arguably provides adequate information on all pRF parameters.

### Voxel Selection

In the present study voxels were treated as subjects in the statistical analyses. Since it is only meaningful to include those voxels that encode visual information it was necessary to separate visual from non-visual voxels for each subject and scan session. This was done in two steps. First, voxels were sorted into two clusters by performing k-means clustering on their fit values for each stimulus configuration (i.e. experimental run) individually. This led to a cluster of visual voxels per experimental run. In the second step only voxels which were consistently placed into the visual cluster for all runs were retained in the analyses. In the first session 2655, 2669, and 1969 voxels were retained for the three subjects, respectively. In the second session 2592, 2764, and 1673 voxels were retained for subjects one, two, and three respectively. In order to check the validity of this data-driven voxel selection procedure we changed the first step to labeling all voxels visual whose Fit values are larger than the 95^th^, 97.5^th^, and 99^th^ percentile. After the second step, the final voxel selection for all three percentiles was identical with that derived using the original procedure.

### Simulated Data

In addition to the empirical data set, we simulated fMRI data from known receptive fields. Specifically, the simulated data was given by
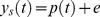
(9)where *p(t)* is the predicted BOLD signal, and *e* is an error term obtained from the residuals of the pRF estimation of subject 1. The BOLD signal *p(t)* was given by applying equations 2 through 7 using a pre-defined set of receptive field parameters, the effective stimulus settings for the wedge, ring, and bar stimuli we previously used in the experimental setup of subject 2, and a prototypical hemodynamic response obtained from BOLD signal of subject 3. The set of pre-defined pRF parameters consists of 36000 parameter configurations each of which corresponds exactly to a point on the search grid. For this set of simulated data all assumptions regarding the pRF model were met while at the same time we searched the parameter space optimally. Therefore, all deviations of estimated pRFs with respect to the true underlying pRFs reflect on the aptness of the stimulus rather than on model choice and parameter estimation procedures.

### Metrics

In order to evaluate the estimation framework as well as the extent to which population receptive fields are consistent over time we calculated a set of metrics. The first of these, exclusively used to evaluate the estimation framework for empirical data, was explained variance. Specifically, we quantified the variance of the measured BOLD signal *y_m_* that was explained by the predicted BOLD signal *y_p_* as the squared Pearson correlation between *y_m_* and *y_p_*.

The second metric, used to evaluate the estimation framework for simulated data as well as to measure temporal consistency of pRFs, was the similarity *S* between model parameters of two receptive fields (of the same voxel but obtained using different parameter sets) *a* and *b* given by



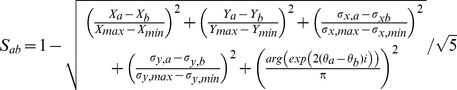
(10)


The differences between pRFs were normalized for each parameter with respect to its range in order to account for the different scales of the parameters and hence to obtain a similarity metric for which every parameter carries equal weight. Additionally, for angles of elongation we calculated an angular difference since an angle of 0 and an angle of π are identical with respect to pRF shape. The factor of two in the angular difference accounts for the fact that the range from 0 to π needs to be stretched around the entire circle for the formula to apply.

Finally, another metric of temporal consistency of pRFs was the correlation of Polar Angle and Eccentricity maps across sessions. For Eccentricity maps this was simply the Pearson correlation coefficient. Since Polar Angle values are circular values, we used the circular correlation coefficient *r*_circ_ proposed by Fisher and Lee [14] for Polar Angle maps:
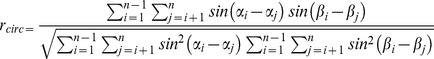
(11)where n is the number of data points and α and β are angles.

### Analyses and Information Aggregation

To establish which choices with regard to the estimation framework are most optimal a ranking procedure based on the Tideman method was employed [15]. Specifically, in order to, for instance, establish which combination of stimulus and mode of presentation should be used, all possible combinations of these choices were pairwise compared to each other with respect to the relevant outcome metric. This was done per subject per session such that each subject casted two votes (one for each session) as to which member of a pair is preferable. The votes were Hedges’ g [16] values which provide a standardized distance between means. Specifically, Hedges’ g is given by



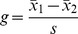
(12)where 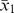
and 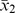 are the sample means and the *s* the pooled standard deviation given by
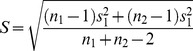
(13)with *s*_1_ and *s*_2_ being the standard deviations and *n*_1_ and *n*_2_ the sample sizes of the two samples, respectively. We created 95% percent confidence intervals around the Hedges’ g values and considered a member of a pair to be preferable to the other only if zero did not fall within this confidence interval. Hence, a vote for one member of a pair was cast if it produced a significantly higher value on the outcome metric than its rival and the vote was weighted by the actual value of Hedges’ g to ensure that strong differences between members carry more weight than weak, albeit significant, differences. The pairwise Hedges’ g values of all subjects and sessions were added up to obtain a matrix of pairwise voting results. The Tideman method [15] was then applied to this matrix of voting results in three steps. 1) Tally: for each pair (A,B) in the matrix the sum of Hedges’ g values for *A* winning over *B* was compared to the sum of Hedges’ g values for *B* winning over *A*. The larger of the two sums represented a majority and hence determined the pair’s winner while the smaller of the two sums represented a minority. 2) Sort: the pairs were ordered based on their majorities with larger majorities preceding smaller ones. In case the winners of two pairs had equal majorities, the pair with the smaller minority was ranked first. 3) Lock: a directed graph in the form of an adjacency matrix was constructed from these pairs. In an adjacency matrix each present connection (edge) between a pair of candidates (vertices) is represented by 1 while an absent connection is represented by 0. Here, edges were drawn from a pair’s winner to a pair’s loser with circular connections omitted. The indegree (number of incoming connections) of each vertex determines its rank with a lower indegree corresponding to a higher rank. Consequently the source of the graph (the vertex with an indegree of zero) is the overall winner.

This procedure was employed for both empirical as well as simulation results. Hedges’ g measures of effect sizes rather than pairwise t-tests were used for two reasons. Firstly, all statistical tests have tremendous power since voxels constitute our sample and the associated sample size renders even negligible effects highly significant. This is especially true for analyses performed on empirical data. Secondly, effect sizes are more informative with respect to best practice in an experimental setup as they provide a straightforward mode of comparison [17].

## Results

### Optimization of the Estimation Framework

#### Stimulus Type and Stimulus Presentation

The first ranking was performed to identify which combination of stimulus type and mode of presentation yields a set of pRF parameters which can predict BOLD signals obtained from different experimental setups measured during the same scanning session. This is to ensure that an estimation framework does not yield stimulus-specific population receptive field estimates. From each set of pRF parameters obtained from a specific combination of stimulus and mode of presentation we predicted BOLD signals for those experimental runs during which another stimulus was presented. Subsequently, we calculated the proportion of variance observed in the measured BOLD signals that was explained by those predictions. The explained variance values obtained for every predicted run were averaged to obtain a single value per voxel. For instance, if parameters were obtained using the bar stimulus, BOLD signals for both the wedge and ring (using both presentation modes) were predicted from the parameter set of each voxel and the explained variance values obtained for the resulting predictions pertaining to wedge and ring were averaged. From these explained variances we derived votes in the form of Hedges’ g measures of effect size (see methods for details). Table 1 shows the voting results for all pairwise comparisons of stimulus-presentation combinations with every voting result indicating the sum of Hedges’ g values for the one-sided comparisons of row larger than column.

**Table 1:** Voting Results for Within Session Explanatory Power.

		WR_pre_		WR_post_		Bar	
		orderly	random	orderly	random	random	random
WR_pre_	orderly		**0.5**	**0.4**	**0.3**	**0.2**	**0.2**
	random	**0.6**		**1.1**	**0.8**	**0.4**	**0.4**
WR_post_	orderly	**0.0**	**0.5**		**0.0**	**0.2**	**0.2**
	random	**0.3**	**0.7**	**0.5**		**0.2**	**0.2**
Bar	orderly	**1.1**	**1.2**	**1.6**	**1.2**		**0.0**
	random	**1.6**	**1.6**	**2.0**	**1.5**	**0.3**	

Each cell represents the sum of hedges’ g values for the pairwise comparison of row over column obtained for each combination of subject and session. Since three subjects were tested in two sessions (each subject casted two votes), there were six votes in total. Only significant results were summed, i.e. those hedges’ g values whose confidence intervals did not include zero. If zero lay within the confidence interval the vote was counted as indifferent between the two options.

Applying the Tideman method [15] to these voting results, we considered each pairwise comparison in turn and determined its winner (tally). Subsequently, we ordered the pairs from largest to smallest majority (sort). Table 2 shows a summary of these steps. Finally, the results were locked in a directed graph (lock). The adjacency matrix of this graph as well as each pair’s indegree is represented in table 3. The randomly presented bar was the source of the graph and thus constituted the top of the hierarchy. The orderly presented bar was ranked second with an indegree of 1, followed by randomly presented WR_pre_ with an indegree of 2. The fourth rank was shared by orderly presented WR_pre_ and randomly presented WR_post_ which both had an indegree of 3. Finally, orderly presented WR_post_ was ranked lowest with an indegree of 5.

**Table 2:** Pairwise Comparisons for Within Session Explanatory Power.

Pair	Winner	Order
WR_pre_, orderly (***g = 0.5***) vs. WR_pre_, random (***g = 0.6***)	WR_pre_, random	**11**
WR_pre_, orderly (***g = 0.4***) vs. WR_post_, orderly (***g = 0***)	WR_pre_, orderly	**13**
WR_pre_, orderly (***g = 0.3***) vs. WR_post_, random (***g = 0.3***)	tie	**15**
WR_pre_, orderly (***g = 0.2***) vs. Bar, orderly (***g = 1.1***)	Bar, orderly	**8**
WR_pre_, orderly (***g = 0.2***) vs. Bar, random (***g = 1.6***)	Bar, random	**2**
WR_pre_, random (***g = 1.1***) vs. WR_post_, orderly (***g = 0.5***)	WR_pre_, random	**9**
WR_pre_, random (***g = 0.8***) vs. WR_post_, random (***g = 0.7***)	WR_pre_, random	**10**
WR_pre_, random (***g = 0.4***) vs. Bar, orderly (***g = 1.2***)	Bar, orderly	**7**
WR_pre_, random (***g = 0.4***) vs. Bar, random (***g = 1.6***)	Bar, random	**4**
WR_post_, orderly (***g = 0***) vs. WR_post_, random (***g = 0.5***)	WR_post_, random	**12**
WR_post_, orderly (***g = 0.2***) vs. Bar, orderly (***g = 1.6***)	Bar, orderly	**3**
WR_post_, orderly (***g = 0.2***) vs. Bar, random (***g = 2***)	Bar, random	**1**
WR_post_, random (***g = 0.2***) vs. Bar, orderly (***g = 1.2***)	Bar, orderly	**6**
WR_post_, random (***g = 0.2***) vs. Bar, random (***g = 1.5***)	Bar, random	**5**
Bar, orderly (***g = 0***) vs. Bar, random (***g = 0.3***)	Bar, random	**14**

The first column shows the comparison of each pair (A,B) including the hedges’ g values of A winning over B (left) as well as B winning over A (right). The second column shows the winner of each pair. Finally, the third column shows the order in which pairs were locked based on the majority starting with the largest.

**Table 3:** Directed Graph for Within Session Explanatory Power.

		WR_pre_		WR_post_		Bar	
		orderly	random	orderly	random	orderly	random
WR_pre_	orderly	**0**	**0**	**1**	**0**	**0**	**0**
	random	**1**	**0**	**1**	**1**	**0**	**0**
WR_post_	orderly	**0**	**0**	**0**	**0**	**0**	**0**
	random	**0**	**0**	**1**	**0**	**0**	**0**
Bar	orderly	**1**	**1**	**1**	**1**	**0**	**0**
	random	**1**	**1**	**1**	**1**	**1**	**0**
Indegree		**3**	**2**	**5**	**3**	**1**	**0**

The graph depicts binary edges leading from row to column as well as the sum total of incoming edges for each vertex (stimulus-presentation combination).

Based on the rankings of the combinations of stimulus type and mode of presentation we derived separate matrices of voting results for stimulus type and mode of presentation. To determine the votes of *A* winning over *B,* where *A* and *B* are different stimulus types, we examined how often the orderly variant of *A* was ranked above both variants of *B* and how often the random variant of *A* was ranked above both variants of *B*. For instance, the orderly variant of WR_pre_ was ranked only above the orderly variant of WR_post_ while the random variant of WR_pre_ was ranked above both variants of WR_post_. This yields a total of three instances where WR_pre_ was ranked above WR_post_. Table 4 shows the voting results obtained accordingly for all stimulus pairs.

**Table 4:** Stimulus Voting Results for Within Session Explanatory Power.

	WR_pre_	WR_post_	Bar
WR_pre_		**3**	**0**
WR_post_	**0**		**0**
Bar	**4**	**4**	

Each cell represents the sum of rankings where the row stimulus was ranked above the column stimulus.

Similarly, to determine the votes of *A* winning over *B,* where *A* and *B* are different modes of presentation, we examined how often each stimulus presented in variant *A* was ranked above all stimuli presented in variant *B*. Table 5 shows the voting results for the comparison of orderly and random presentations and allows inference of the Condorcet winner. A candidate in an election is considered the Condorcet winner if that candidate wins by majority rule against each other candidate. This condition to determine a winner was developed by the Marquis de Condorcet in 1785 [21]. Since the bar was ranked consistently above the other stimuli, it constituted the Condorcet winner in the context of predicting BOLD activity obtained from other retinotopic mapping stimuli. Since in the majority of rankings the random presentation variant was preferable to the orderly presentation variant, it constituted the Condorcet winner in the context of predicting BOLD activity of other stimuli.

**Table 5:** Mode of Presentation Voting Results for Within Session Explanatory Power.

	Orderly	Random
Orderly		**2**
Random	**6**	

Each cell represents the sum of rankings where the mode of presentation given by the row was ranked above the mode of presentation given by the column.

The second ranking was performed in order to identify which combination of stimulus and mode of presentation yields a set of pRF parameters from which a BOLD signal measured at a different point in time can be predicted most accurately. To this end we again calculated the proportion of variance observed in the measured BOLD signal that a model BOLD signal based on pRF parameters from a specific combination can explain. In contrast to the previous analysis, however, the pRF parameters were obtained in one session while the measured BOLD signal was taken from a second session.

Table 6 shows the voting results for the variance explained across sessions (tally). By applying the Tideman method [15] we obtained the winners of each pairwise comparison as well as their orderings (sort). The comparisons, winners, and orderings are given in table 7. Subsequently, we obtained the ranking of the combinations by locking the results in a directed graph (lock; see table 8). The randomly presented bar was the source of the graph and thus constituted the top of the hierarchy. The orderly presented bar was ranked second with an indegree of 1, followed by randomly presented WR_pre_ with an indegree of 2. WR_post_ presented randomly was ranked fourth with an indegree of 3. The fifth rank was occupied by orderly presented WR_pre_ with an indegree of 4. Finally, orderly presented WR_post_ was ranked lowest with an indegree of 5.

**Table 6:** Voting Results for Across Session Explanatory Power.

		WR_pre_		WR_post_		Bar	
		orderly	random	orderly	random	orderly	random
WR_pre_	orderly		**0.4**	**0.3**	**0.2**	**0.3**	**0.1**
	random	**0.5**		**0.9**	**0.7**	**0.5**	**0.3**
WR_post_	orderly	**0.0**	**0.4**		**0.0**	**0.2**	**0.1**
	random	**0.3**	**0.5**	**0.4**		**0.4**	**0.2**
Bar	orderly	**0.9**	**1.0**	**1.3**	**1.0**		**0.0**
	random	**1.2**	**1.2**	**1.5**	**1.3**	**0.3**	

Each cell represents the sum of hedges’ g values for the pairwise comparison of row over column obtained for each combination of subject and session. Since three subjects were tested in two sessions (each subject casted two votes), there were six votes in total. Only significant results, that is, those hedges’ g values whose confidence intervals did not include zero, were summed. If zero lay within the confidence interval the vote was counted as indifference between the two options.

**Table 7:** Pairwise Comparisons for Across Session Explanatory Power.

Pair	Winner	Order
WR_pre_, orderly (***g = 0.4***) vs. WR_pre_, random (***g = 0.5***)	WR_pre_, random	**11**
WR_pre_, orderly (***g = 0.3***) vs. WR_post_, orderly (***g = 0***)	WR_pre_, orderly	**13**
WR_pre_, orderly (***g = 0.2***) vs. WR_post_, random (***g = 0.3***)	WR_post_, random	**15**
WR_pre_, orderly (***g = 0.3***) vs. Bar, orderly (***g = 0.9***)	Bar, orderly	**8**
WR_pre_, orderly (***g = 0.1***) vs. Bar, random (***g = 1.2***)	Bar, random	**4**
WR_pre_, random (***g = 0.9***) vs. WR_post_, orderly (***g = 0.4***)	WR_pre_, random	**9**
WR_pre_, random (***g = 0.7***) vs. WR_post_, random (***g = 0.5***)	WR_pre_, random	**10**
WR_pre_, random (***g = 0.5***) vs. Bar, orderly (***g = 1***)	Bar, orderly	**7**
WR_pre_, random (***g = 0.3***) vs. Bar, random (***g = 1.2***)	Bar, random	**5**
WR_post_, orderly (***g = 0***) vs. WR_post_, random (***g = 0.4***)	WR_post_, random	**12**
WR_post_, orderly (***g = 0.2***) vs. Bar, orderly (***g = 1.3***)	Bar, orderly	**2**
WR_post_, orderly (***g = 0.1***) vs. Bar, random (***g = 1.5***)	Bar, random	**1**
WR_post_, random (***g = 0.4***) vs. Bar, orderly (***g = 1***)	Bar, orderly	**6**
WR_post_, random (***g = 0.2***) vs. Bar, random (***g = 1.3***)	Bar, random	**3**
Bar, orderly (***g = 0***) vs. Bar, random (***g = 0.3***)	Bar, random	**14**

The first column shows the comparison of each pair (A,B) including the hedges’ g values of A winning over B (left) as well as B winning over A (right). The second column shows the winner of each pair. Finally, the third column shows the order in which pairs were locked based on the majority starting with the largest.

**Table 8:** Directed Graph for Across Session Explanatory Power.

		WR_pre_		WR_post_		Bar	
		orderly	random	orderly	random	orderly	random
WR_pre_	orderly	**0**	**0**	**1**	**0**	**0**	**0**
	random	**1**	**0**	**1**	**1**	**0**	**0**
WR_post_	orderly	**0**	**0**	**0**	**0**	**0**	**0**
	random	**1**	**0**	**1**	**0**	**0**	**0**
Bar	orderly	**1**	**1**	**1**	**1**	**0**	**0**
	random	**1**	**1**	**1**	**1**	**1**	**0**
Indegree		**4**	**2**	**5**	**3**	**1**	**0**

The graph depicts binary edges leading from row to column as well as the sum total of incoming edges for each vertex (stimulus-presentation combination).

Based on the rankings of the combinations of stimulus type and stimulus presentation we derived matrices of voting results for type and presentation, respectively. Table 9 shows the voting results with regard to stimulus. Since the bar was ranked consistently above the other stimuli, it constituted the Condorcet winner in the context of predicting BOLD activity across sessions.

**Table 9:** Stimulus Voting Results for Across Session Explanatory Power.

	WR_pre_	WR_post_	Bar
WR_pre_		**3**	**0**
WR_post_	**1**		**0**
Bar	**4**	**4**	

Each cell represents the sum of rankings where the row stimulus was ranked above the column stimulus.

With regard to stimulus presentation, table 10 shows the voting results for the comparison of orderly and random presentations. Since for the majority of rankings the random presentation variant was preferable to the orderly presentation variant, it constituted the Condorcet winner in the context of predicting BOLD activity across sessions.

**Table 10:** Mode of Presentation Voting Results for Across Session Explanatory Power.

	Orderly	Random
Orderly		**2**
Random	**7**	

Each cell represents the sum of rankings where the mode of presentation given by the row was ranked above the mode of presentation given by the column.

#### Stimulus Type and Continuance

The third ranking was performed for simulated data with the aim to investigate which stimulus type either interspersed by mean luminance (ML) periods or not (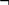
ML) most faithfully recovers known population receptive field parameters. To this end we obtained the similarity between original and recovered pRF parameters. Since neither attention nor lateral connectivity was modeled in our simulations all stimuli were presented in an orderly fashion only. However, each stimulus was presented both with and without mean luminance periods in two separate runs. The presence of mean luminance periods has been argued to improve the estimation of receptive field parameters, especially if receptive fields are large [1]. As with the empirical results rankings are performed on the Hedges’ g values obtained for the pairwise comparison of all combinations of stimulus and mean luminance. Table 11 shows the resulting votes.

**Table 11:** Voting Results for pRF Estimate Similarity.

		WR_pre_		WR_post_		Bar	
		ML	¬ML	ML	¬ML	ML	¬ML
WR_pre_	ML		**2.1**	**1.6**	**1.9**	**0.0**	**0.0**
	¬ML	**0.0**		**0.0**	**0.0**	**0.0**	**0.0**
WR_post_	ML	**0.0**	**0.0**		**0.0**	**0.0**	**0.0**
	¬ML	**0.0**	**0.0**	**0.0**		**0.0**	**0.0**
Bar	ML	**1.0**	**3.1**	**2.4**	**2.8**		**0.0**
	¬ML	**0.0**	**2.6**	**2.0**	**2.3**	**0.0**	

Each cell represents the sum of hedges’ g values for the pairwise comparison of row over column obtained for simulation results of each condition. Only significant results, that is, those hedges’ g values whose confidence intervals did not include zero, were summed. If zero lay within the confidence interval the vote was counted as indifference between the two options.

Applying the Tideman method [15] we obtained the winners of each pairwise comparison as well as their orderings (tally). The comparisons, winners, and orderings are given in table 12. Subsequently, we obtained the ranking of the combinations by locking the results in a directed graph (lock; see table 13). The resulting graph had two sources since both versions of the bar, that is presented interspersed and not interspersed by periods of mean luminance, had only outgoing but no incoming connections. With an indegree of 1 WR_pre_ presented interspersed with mean luminance periods was ranked second. Finally, all remaining combinations of stimulus and presence or absence of mean luminance periods had an indegree of 3 and thus constitute equally undesirable choices.

**Table 12:** Pairwise Comparisons for pRF Estimate Similarity.

Pair	Winner	Order
WR_pre_, ML (***g = 2.1***) vs. WR_pre_, ¬ML (***g = 0***)	WR_pre_, ML	**6**
WR_pre_, ML (***g = 1.6***) vs. WR_post_, ML (***g = 0***)	WR_pre_, ML	**8**
WR_pre_, ML (***g = 1.9***) vs. WR_post_, ¬ML (***g = 0***)	WR_pre_, ML	**7**
WR_pre_, ML (***g = 0***) vs. Bar, ML (***g = 1***)	Bar, ML	**9**
WR_pre_, ML (***g = 0***) vs. Bar, ¬ML (***g = 0***)	Tie	**10**
WR_pre_, ¬ML (***g = 0***) vs. WR_post_, ML (***g = 0***)	Tie	**11**
WR_pre_, ¬ML (***g = 0***) vs. WR_post_, ¬ML (***g = 0***)	Tie	**12**
WR_pre_, ¬ML (***g = 0***) vs. Bar, ML (***g = 3.1***)	Bar, ML	**1**
WR_pre_, ¬ML (***g = 0***) vs. Bar, ¬ML (***g = 2.6***)	Bar, ¬ML	**3**
WR_post_, ML (***g = 0***) vs. WR_post_, ¬ML (***g = 0***)	Tie	**13**
WR_post_, ML (***g = 0***) vs. Bar, ML (***g = 2.4***)	Bar, ML	**4**
WR_post_, ML (***g = 0***) vs. Bar, ¬ML (***g = 2***)	Bar, ¬ML	**14**
WR_post_, ¬ML (***g = 0***) vs. Bar, ML (***g = 2.8***)	Bar, ML	**2**
WR_post_, ¬ML (***g = 0***) vs. Bar, ¬ML (***g = 2.3***)	Bar, ¬ML	**5**
Bar, ML (***g = 0***) vs. Bar, ¬ML (***g = 0***)	Tie	**15**

The first column shows the comparison of each pair (A,B) including the hedges’ g values of A winning over B (left) as well as B winning over A (right). The second column shows the winner of each pair. Finally, the third column shows the order in which pairs were locked based on the majority starting with the largest.

**Table 13:** Directed Graph for pRF Estimate Similarity.

		WR_pre_		WR_post_		Bar	
		ML	¬ML	ML	¬ML	ML	¬ML
WR_pre_	ML	**0**	**1**	**1**	**1**	**0**	**0**
	¬ML	**0**	**0**	**0**	**0**	**0**	**0**
WR_post_	ML	**0**	**0**	**0**	**0**	**0**	**0**
	¬ML	**0**	**0**	**0**	**0**	**0**	**0**
Bar	ML	**1**	**1**	**1**	**1**	**0**	**0**
	¬ML	**0**	**1**	**1**	**1**	**0**	**0**
Indegree		**1**	**2**	**2**	**2**	**0**	**0**

The graph depicts binary edges leading from row to column as well as the sum total of incoming edges for each vertex (stimulus - mean luminance combination).

Based on the rankings of the combinations of stimulus type and continuance we derived matrices of voting results for type and continuance, respectively. Table 14 shows the voting results with regard to stimulus. Since the bar was ranked consistently above the other stimuli, it constituted the Condorcet winner for the simulations.

**Table 14:** Stimulus Voting Results for pRF Estimate Similarity.

	WR_pre_	WR_post_	Bar
WR_pre_		**2**	**0**
WR_post_	**0**		**0**
Bar	**4**	**4**	

Each cell represents the sum of rankings where the row stimulus was ranked above the column stimulus.

With regard to continuance, table 15 shows the voting results for the comparison of presentations with and without interspersed mean luminance periods. Since for the majority of rankings the presence of mean luminance periods was preferable to their absence, the inclusion of mean luminance periods constituted the Condorcet winner for the simulations.

**Table 15:** Mean Luminance Voting Results for pRF Estimate Similarity.

	ML	¬ML
ML		**4**
¬ML	**2**	

Each cell represents the sum of rankings where the continuance (absence or presence of mean luminance periods) given by the row was ranked above the continuance given by the column.

The beneficial effect of mean luminance periods is supposed to manifest itself mainly for late visual areas. For this reason, we repeated the current ranking for V1 and V4 type receptive fields separately. To create V1 and V4 type receptive fields we used two different sets of receptive field sizes while keeping the remaining parameters constant. The sizes were a linear function of the eccentricity with biologically realistic slopes. Specifically, the slope for V4 (.84) was much steeper than the slope for V1 (.21) [18]. We concentrate here only on the main findings of these additional rankings and provide detailed analysis in appendices S1 and S2. As expected for V1 type receptive fields, neither stimulus choice nor the inclusion of mean luminance periods had strong effects on pRF estimation performance. Nonetheless, the effect of mean luminance is opposite to what has been proposed for late visual areas. Namely, omission of mean luminance periods leads to slightly better pRF estimates than their inclusion. With regard to stimulus choice, WR_post_ and bar both ranked above WR_pre_ but were indistinguishable among themselves (for a detailed analysis see appendix S1). For V4 the general findings of our third ranking reappear. With regard to stimuli, the bar was superior to both WR_pre_ and WR_post_ with WR_pre_, in turn, being superior to WR_post_. With regard to the mean luminance periods, it was clearly beneficial to include such periods in pRF estimation (for a detailed analysis see appendix S2). These findings support the notion that mean luminance periods aid the estimation of visual areas with large receptive field sizes.

#### Data Preparation

**Figure 3:**
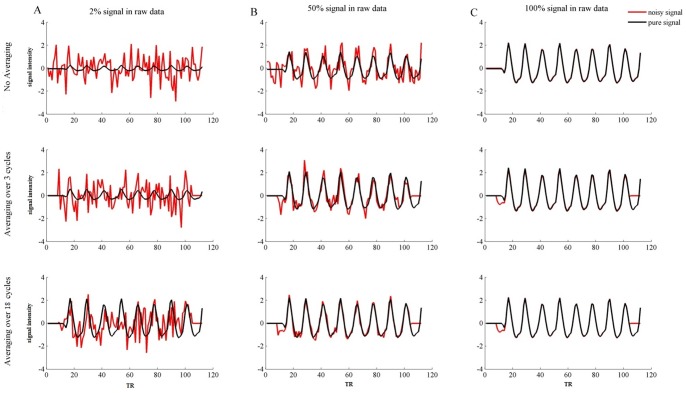
Averaged and non-averaged BOLD signals for different noise levels. This figure shows both averaged and non-averaged versions of BOLD signals for three levels of noise for one simulated voxel. The red line indicates the overall BOLD signal resulting from stimulation as well as additive noise. Note that the baseline period (8 TRs) before and after the stimulation period are set to zero. The black line indicates the signal purely as a function of stimulation contained within the larger BOLD signal. Column A) shows non-averaged signal (upper row), signal averaged over 3 cycles (middle row), and signal averaged over 20 cycles (lower row) for raw (i.e. not averaged) BOLD of which 2 percent is stimulus-related signal. More extensive averaging reduces noise to a greater extent and hence increases the stimulus-related variation in the overall signal. Specifically, only for signal averaged over 20 cycles does the overall signal resemble the purely stimulation related signal. Column B) shows non-averaged signal (upper row), signal averaged over 3 cycles (middle row), and signal averaged over 20 cycles (lower row) for raw BOLD of which 50 percent is stimulus-related signal. More extensive averaging reduces noise to a greater extent and hence increases the stimulus-related variation in the overall signal. Nonetheless, even for non-averaged data, the overall signal bears resemblance to the purely stimulus-related signal. Column C) shows non-averaged signal (upper row), signal averaged over 3 cycles (middle row), and signal averaged over 20 cycles (lower row) for raw BOLD of which 100 percent is stimulus-related signal. Since there is no noise in the data, averaging has no effect.

A final consideration pertains to the advantages of performing pRF estimation on BOLD signal averaged across several stimulus cycles. To this end we compared the explained variance as well as Similarity among pRF parameters for estimated and known underlying population receptive fields for averaged and non-averaged simulated BOLD signals. The simulated data came from a procedure using a bar stimulus presented in an orderly fashion without mean luminance gaps. This estimation framework was chosen since it represents straightforward matches of stimulus positions from one cycle to the next. Three different conditions were considered. In the first, three repetitions of the 8 orientation (4) and direction (2) combinations were used to generate a non-averaged simulated BOLD signal. In the second, the BOLD signal resulting from these three repetitions was averaged. Finally, in the third condition, BOLD signal was generated from 18 stimulus repetitions and subsequently averaged. Additionally, we parametrically varied the amount of additive noise so as to investigate the advantage that averaging conveys for hardly noisy to very noisy data sets. Figure 3 shows both averaged and non-averaged BOLD signals for different noise levels. The red lines indicate the signal including noise while the black lines indicate the pure signal resulting from stimulation contained within the noisy signal. Especially for noisy signals, i.e. where signal resulting from stimulation constitutes merely 2 percent of the resulting BOLD signal (Figure 3A), averaging lead to profound increases in stimulation related variance contained in the overall noisy signal. Population receptive field parameters were estimated from averaged as well as non-averaged signals for each level of noise.

**Figure 4:**
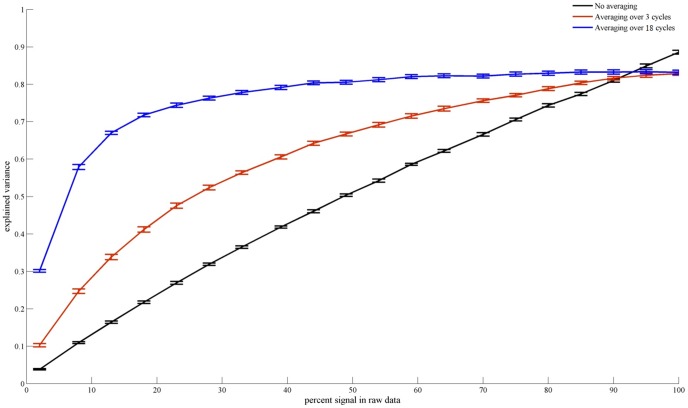
Explained variance within averaged and non-averaged BOLD signals for different noise levels. This figure shows the amount of variance explained for averaged and non-averaged BOLD signals based on pRF parameters estimated from these signals as a function of the percent stimulus-related signal in the raw data. The explained variance for non-averaged signals (black line) scales linearly with the percentage of stimulus-related signal in the raw data. That is, the estimated pRF parameters explain as much variance of the overall signal as is actually due to stimulation. For averaged signals, the amount of variance explained both starts off higher and reaches a plateau faster than for the non-averaged signal. This effect is stronger for signal averaged across 20 cycles (blue line) than for signal averaged across 3 cycles (red line).

Figure 4 shows the variance explained by a predicted BOLD signal where the prediction is based on pRF parameters estimated from non-averaged signals (upper row), signals averaged over 3 cycles (middle row), and signals averaged over 18 cycles (lower row), respectively. As expected from the higher stimulation-related signal content for averaged signals, averaging had a tremendous benefit on the explained variance. Additionally, more averaging resulted in larger proportions of explained variance.

**Figure 5:**
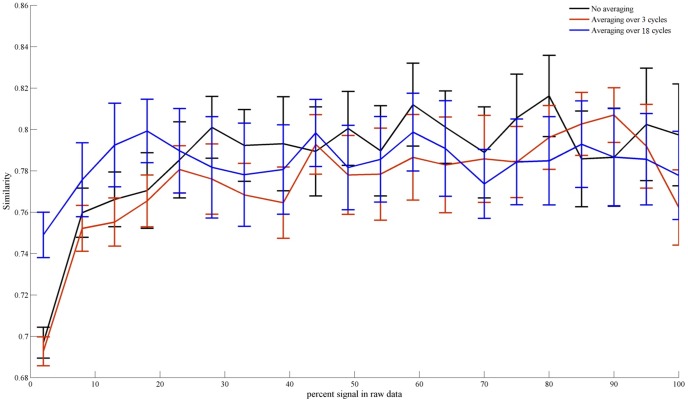
Similarity between known and estimated pRF parameters for averaged and non-averaged BOLD signals at different noise levels. The figure shows the Similarity between known and estimated pRF parameters for averaged and non-averaged BOLD signals as a function of the percent stimulus-related signal in the raw data. For extreme noise, where stimulus-related signal constitutes only 2 percent of the overall signal, averaging over 20 cycles (blue line) produces higher Similarity values than either non-averaged signal (black line) or signal averaged over 3 cycles (red line). For larger percentages of stimulus-related signal within the overall signal, averaging shows no effect.

Figure 5 shows the Similarity between the true underlying pRF parameters and those estimated from the simulated BOLD signals. For very noisy data, i.e. where signal resulting from stimulation constitutes merely 2 percent of the resulting BOLD signal (Figure 3A), estimation from signal averaged over 18 cycles showed significantly larger Similarity values than estimation from both non-averaged signal and signal averaged over 3 cycles. For larger percentages of signal due to stimulation, however, the beneficial effect of extensive averaging disappeared. Following up on this result we investigated the effect of averaging in more detail for BOLD signals of which only 2 percent are due to averaging. To this end we varied the number of cycles over which averaging occurs in the range from 3 to 18 (in steps of 3). This analysis revealed that performance improved as averaging was performed for 3 to 9 cycles with mean Similarity values of µ = .69 [.68.70], µ = .72 [.71.73], and µ = .75 [.74.76] for 3, 6 and 9 cycles, respectively. Beyond 9 cycles averaging had no further beneficial effect with a mean Similarity value of µ = .75 [.74.76] for signals averaged over 18 cycles.

### Consistency

**Figure 6:**
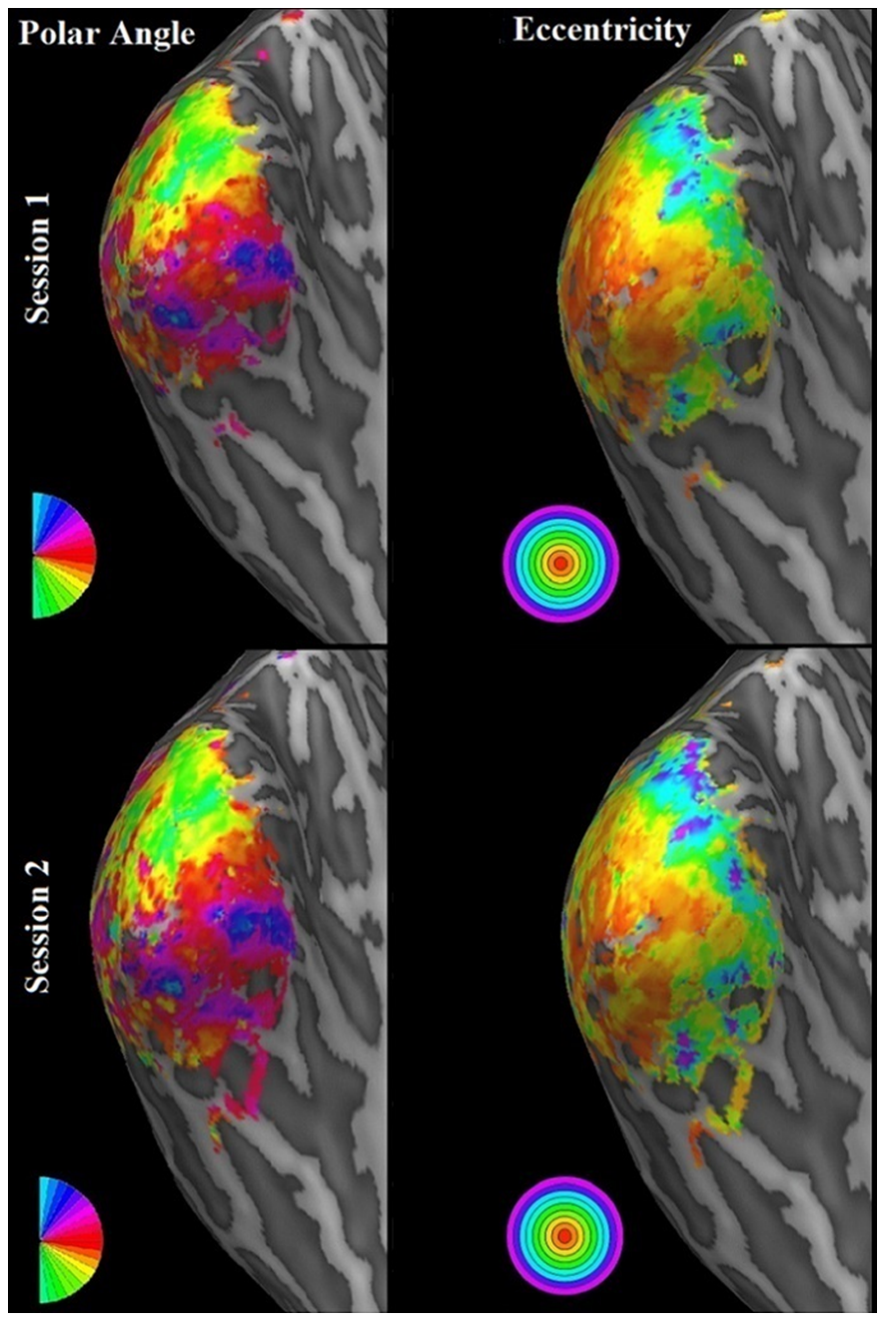
Retinotopy Surface Maps. This figure shows exemplary polar angle as well as eccentricity maps of subject 3. The maps were obtained from a randomly presented bar stimulus within two separate sessions. The upper row shows maps of the first session while the lower row shows maps of session two. In accordance with the correlation results between maps (see Figure 8 for details), the two polar angle and eccentricity maps are highly visually similar.

**Figure 7:**
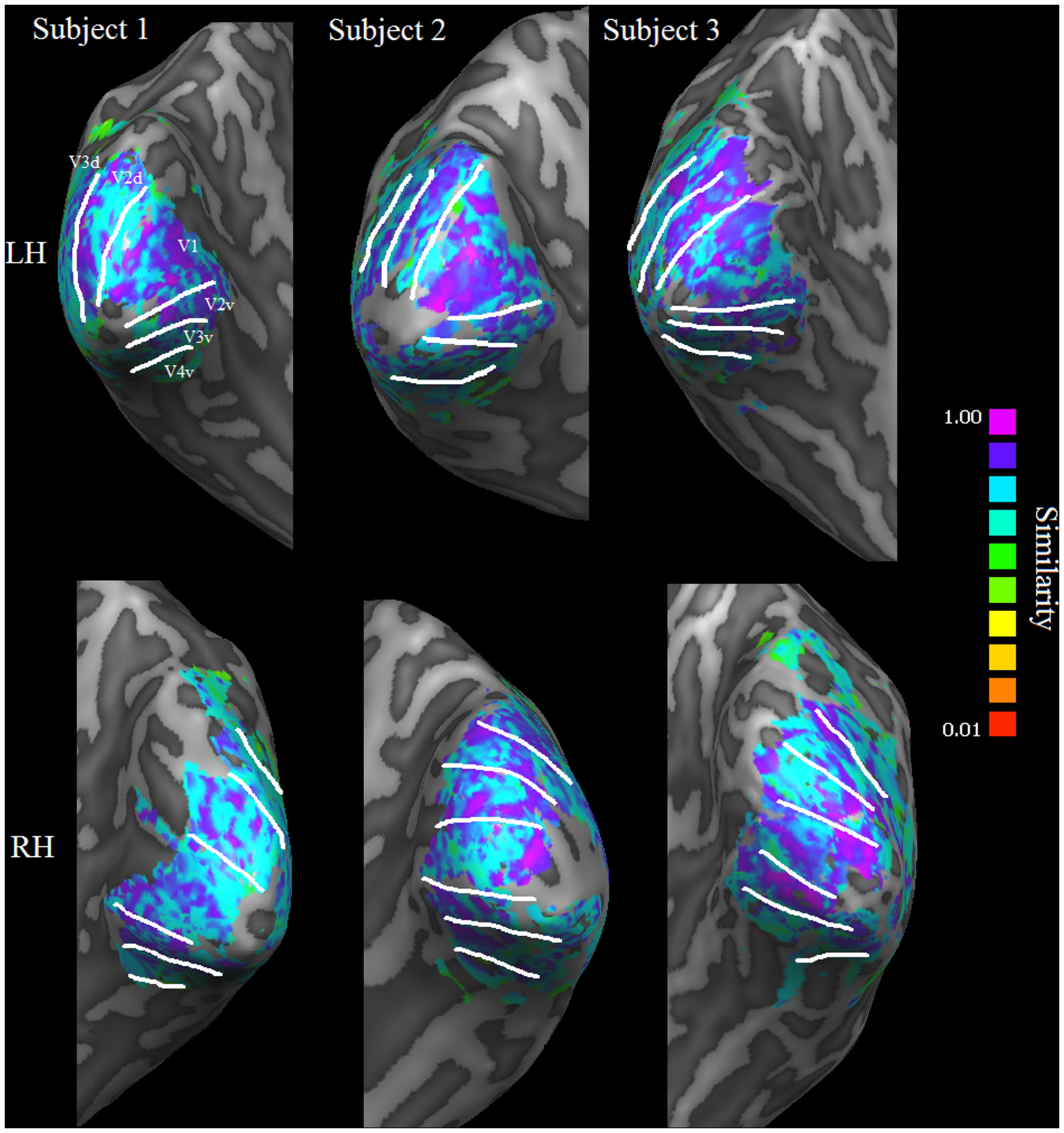
Similarity Surface Maps. This figure shows Similarity between pRF estimates obtained in sessions 1 and 2 for all three participants. The maps were obtained from a randomly presented bar stimulus within the two separate sessions. The upper row shows maps for the left hemisphere while the lower row shows maps for the right hemisphere. Border delineating visual areas V1, V2, V3, and V4 obtained from the retinopy resulting from this stimulus are superimposed on the maps. The Similarity values show no systematic variation across subjects or along the visual hierarchy.

**Figure 8:**
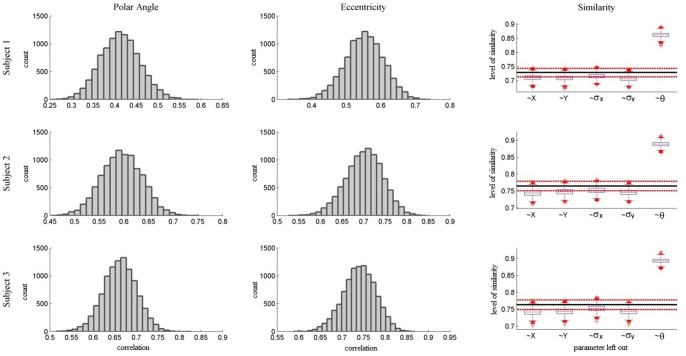
Temporal Consistency. This figure shows the correlation between polar angle and eccentricity maps obtained from two temporally separated sessions as well as the Similarity between pRF parameters estimated from those sessions. The results are shown individually for each subject (rows). For all subjects the distributions of correlations for both polar angle and eccentricity maps obtained using bootstrapping indicate a good fit between the maps. The final column shows Similarity values for all three subjects. The black horizontal line indicates the mean Similarity between the complete set of pRF parameters estimated from the two sessions. The red lines indicate confidence intervals around this mean as obtained via bootstrapping. The boxplots show the Similarity between a series of two sets of pRF parameters each time excluding one parameter in the Similarity calculation. While excluding any location or size parameter has no significant effect on Similarity, removing the angle of elongation significantly increases the Similarity between the two sets. This implies that location and size parameters are estimated very consistently. Angle of elongation, however, is subject to variability.

In addition to stimulation procedure optimization we investigated the consistency of receptive fields across scanning sessions. To this end we correlated Polar Angle and Eccentricity maps obtained from different sessions and calculated the Similarity between pRF parameter estimates of the two sessions. For Polar Angle maps we obtained circular correlations. We restricted our analysis to data obtained from presenting a bar stimulus in a random fashion interspersed with mean luminance periods. Additionally, we only considered voxels which had previously been selected for at least one of the two sessions. The means of all metrics were obtained by bootstrapping. To gain a clear picture which pRF parameters were more or less consistently estimated, we additionally used bootstrapping to obtain means of Similarity values resulting from successively leaving each parameter out. Figure 6 shows exemplary polar angle and eccentricity maps of subject 3 for the two sessions on an inflated cortex mesh while figure 7 shows surface maps detailing the Similarity values between pRF estimates of sessions 1 and 2. Results of the Similarity analyses are finally shown in Figure 8.

**Figure 9:**
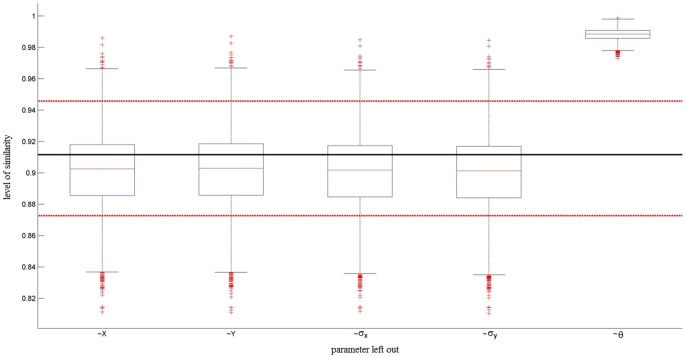
Similarity between known and estimated pRF parameters. This figure shows the Similarity between the entire sets of known and estimated pRF parameters (horizontal lines, black = mean, red = confidence interval obtained via bootstrapping) and the Similarity between a series of sets while each time excluding one parameter (boxplots). While excluding any location or size parameter has no significant effect on Similarity, removing the angle of elongation significantly increases the Similarity between the two sets. This implies that location and size parameters can be estimated very accurately. The angle of elongation parameter, however, diverges somewhat from ground truth.

All metrics indicated high consistency of pRF parameters across sessions. Specifically, polar angle maps showed a circular correlation of r = .41 [.34.49] for subject one, r = .6 [.53.66] for subject two, and r = .66 [.6.73] for subject three. Eccentricity maps showed a correlation of r = .55 [.46.64] for subject one, r = .7 [.63.77] for subject two, and r = .74 [.68.8] for subject three. Finally, mean Similarity was µ = .73 [.71.74], µ = .76 [.75.78], and µ = .75 [.74.77] for subjects one, two, and three, respectively. As can be seen from the third column of Figure 8 removing any of the position or size pRF parameters in the calculation of the Similarity did not significantly affect the result. Leaving out the angle of elongation, however, caused the Similarity values to rise significantly. The mean Similarity of pRF location and size alone, i.e. leaving out angle of elongation, was µ = .86 [.85.88], µ = .89 [.88.9], and µ = .89 [.88.9] for the three subjects, respectively. This implies that while location and size parameter estimates were highly consistent across sessions, the angle of elongation estimate changed somewhat across sessions. To establish whether this was due to dynamic changes in the true underlying receptive fields or simply due to a general difficulty in estimating this parameter we performed bootstrapping on the Similarity values between true pRF parameters and those estimated from simulated data using the same stimulus. The results are shown in Figure 9. These results showed the same overall pattern with highly accurate estimations of location and size parameters but not of the angle of elongation. This implies that the inconsistencies across sessions with regard to that parameter were likely caused by a general difficulty in estimating it rather than undergoing dynamic changes. This result is in line with previous findings of Lee et al. [2] who showed that directly fitting an anisotropic pRF model to fMRI data cannot identify the angle of elongation with an equal degree of precision as for the other parameters.

## Discussion

The present study pursued two goals. One of these goals concerns the identification of best practices with regard to the estimation framework in the context of population receptive field mapping. The other concerns the validity of the assumption that receptive fields are static or temporally consistent. With regard to the former goal our results show that a bar stimulus yields a set of pRF parameters from which BOLD activity resulting from different visual stimulation protocols can be reliably predicted. Additionally, simulation results, for which all assumptions regarding the underlying pRF structure are met by design, show that pRF estimates obtained from stimulation using a bar are more accurate than the estimates obtained from stimulation using a wedge-ring. For these reasons we recommend a bar stimulus. Additionally, the merit of pRF estimates increases for any stimulus, if it is presented in a random fashion. Furthermore, pRF estimation in areas with large receptive fields strongly benefits if stimulation is interspersed with mean luminance periods while estimation of early visual cortex is hardly affected. We, therefore, recommend the use of a bar stimulus presented randomly and interspersed with mean luminance periods for pRF mapping procedures.

With regard to data preparation the benefit of averaging the BOLD signal across stimulus cycles depends on the level of noise in the data. If noise is low, averaging conveys no advantage in terms of the precision with which pRF parameters are estimated even though a larger proportion of variance in the BOLD signal is explained. Only for extremely noisy data; i.e. for very small voxels, can averaging provide more accurate pRF parameter estimates. In order for averaging to take effect, however, a sufficiently large number (∼9) of stimulus cycles is needed. Nonetheless, averaging is accompanied by benefits other than precision in estimation. First of all, the increasing amount of variance that can be explained for averaged as compared to non-averaged signals provides a straightforward demarcation of those voxels processing visual information from those that do not. Another benefit is that averaging the signal reduces the amount of data points and hence reduces the time needed for the estimation process. For these reasons we generally recommend signal averaging and regard it essential for high resolution fMRI data. This requires that the randomization is identical for all stimulus cycles thus limiting the number of possible cycles before the order becomes predictable. An alternative approach might be to concatenate stimulus cycles rather than to average across them. This would allow full randomization and maintain an unpredictable order of stimulus presentations. How such an approach compares to straightforward averaging is an interesting question which remains to be elucidated.

In order to further improve the estimation framework, it is possible to extent stimulation to incorporate different types of aperture content. Usually, stimulus aperture reveals a flickering checkerboard with vertical and horizontal borders. It might be interesting to vary the orientation of these borders in order to accommodate for the radial bias hypothesis [19] and to examine how this affects pRF parameter estimates. Additionally, for the study of pRFs of late visual areas which react primarily to complex visual stimuli it might prove fruitful for the stimulus aperture to reveal complex shapes, motion, or contours [20]. Furthermore, in order to tailor the pRF the estimation framework to more specific research questions such as disease, it might be necessary to further adapt the stimuli. It had previously been shown, for instance, that randomly presented multifocal stimuli improve pRF estimation in the presence of foveal scotomas [9].

Our results with regard to the second goal show that estimated pRF parameters appear to be generally very consistent over time. This is especially true for pRF location and size for which fluctuations in parameter values across sessions are within a very narrow range. With regard to the angle of elongation of anisotropic receptive fields fluctuations in parameter values appear to fluctuate within a somewhat broader range. The consistency of angle of elongation is thus lower than that of other parameters. A possible remedy for this is to employ a pRF mapping approach without a priori assumptions with regard to the pRF shape, as proposed by Lee et al. [2]. This might narrow the range of acceptable values for angle of elongation and consequently boost this parameter’s temporal consistency. Another important methodological consideration is that Similarity values observed empirically are lower than those observed for simulations. This is attributable to the fact that the parameter space was exhaustive for simulations by design while it can hardly be considered to cover the entire range of possible parameter values among voxels. It is thus conceivable that where voxels have an actual receptive field somewhere between two or more points in the examined parameter space it might be attributed to one of them stochastically and thus differ from session to session.

Overall our results suggest that a randomly presented bar stimulus interspersed with mean luminance periods should be used for the estimation of population receptive fields. Ideally but not necessarily, the resulting data should be averaged. Receptive field parameters thus obtained are temporarily stable and can be utilized for numerous applications without hesitation.

## Supporting Information

File S1 **Illustrative visualizations of stimulus presentations for subject one.**
(ZIP)

Appendix S1 **Evaluation of pRF estimation procedures from simulations of V1 type receptive fields.**
(DOCX)

Appendix S2 **Evaluation of pRF estimation procedures from simulations of V4 type receptive fields.**
(DOCX)
