# Supplementary material for: Evaluating Population Receptive Field Estimation Frameworks in Terms of Robustness and Reproducibility
Source: PLoS One. 2014 Dec 2;9(12):e114054. doi: 10.1371/journal.pone.0114054 (PMC4252088; doi:10.1371/journal.pone.0114054)
Supplement: Appendix S2 — Evaluation of pRF estimation procedures from simulations of V4 type receptive fields. (DOCX) [file pone.0114054.s003.docx]

**Appendix S2**: Evaluation of pRF estimation procedures from simulations of V4 type receptive fields.

Here we present the ranking for simulated data with underlying V4 type receptive fields with the aim to investigate which stimulus type either interspersed by mean luminance (ML) periods or not (¬ML) most faithfully recovers the known population receptive field parameters. To this end we obtained the similarity between original and recovered pRF parameters. Each stimulus was presented both with and without mean luminance periods in two separate runs. Appendix S2.1 shows the resulting votes.

**Appendix S2.1: Voting Results for pRF Estimate Similarity**

|  | | WR_pre_ | | WR_post_ | | Bar | |
| --- | --- | --- | --- | --- | --- | --- | --- |
|  |  | ML | ¬ML | ML | ¬ML | ML | ¬ML |
| WR_pre_ | ML |  | **0.0** | **0.4** | **0.5** | **0.0** | **0.0** |
|  | ¬ML | **0.0** |  | **0.0** | **0.3** | **0.0** | **0.0** |
| WR_post_ | ML | **0.0** | **0.0** |  | **0.0** | **0.0** | **0.0** |
|  | ¬ML | **0.0** | **0.0** | **0.0** |  | **0.0** | **0.0** |
| Bar | ML | **0.6** | **0.9** | **1.1** | **1.2** |  | **0.5** |
|  | ¬ML | **0.0** | **0.3** | **0.4** | **0.6** | **0.0** |  |

**Each cell represents the sum of hedges’ g values for the pairwise comparison of row over column obtained for simulation results of each condition. Only significant results, that is, those hedges’ g values whose confidence intervals did not include zero, were summed. If zero lay within the confidence interval the vote was counted as indifference between the two options.**

Applying the Tideman method [15] we obtained the winners of each pairwise comparison as well as their orderings. The comparisons, winners, and orderings are given in Appendix S2.2.

**Appendix S2.2: Pairwise Comparisons for pRF Estimate Similarity**

| Pair | Winner | Order |
| --- | --- | --- |
| WR_pre_, ML (***g = 0***) vs. WR_pre_, ¬ML (***g = 0***) | Tie | **12** |
| WR_pre_, ML (***g = 0.4***) vs. WR_post_, ML (***g = 0***) | WR_pre_, ML | **8** |
| WR_pre_, ML (***g = 0.5***) vs. WR_post_, ¬ML (***g = 0***) | WR_pre_, ML | **6** |
| WR_pre_, ML (***g = 0***) vs. Bar, ML (***g = 0.6***) | Bar, ML | **4** |
| WR_pre_, ML (***g = 0***) vs. Bar, ¬ML (***g = 0***) | Tie | **13** |
| WR_pre_, ¬ML (***g = 0***) vs. WR_post_, ML (***g = 0***) | Tie | **14** |
| WR_pre_, ¬ML (***g = 0.3***) vs. WR_post_, ¬ML (***g = 0***) | WR_pre_, ¬ML | **10** |
| WR_pre_, ¬ML (***g = 0***) vs. Bar, ML (***g = 0.9***) | Bar, ML | **3** |
| WR_pre_, ¬ML (***g = 0***) vs. Bar, ¬ML (***g = 0.3***) | Bar, ¬ML | **11** |
| WR_post_, ML (***g = 0***) vs. WR_post_, ¬ML (***g = 0***) | Tie | **15** |
| WR_post_, ML (***g = 0***) vs. Bar, ML (***g = 1.1***) | Bar, ML | **2** |
| WR_post_, ML (***g = 0***) vs. Bar, ¬ML (***g =* 0.4**) | Bar, ¬ML | **9** |
| WR_post_, ¬ML (***g = 0***) vs. Bar, ML (***g =* 1.2**) | Bar, ML | **1** |
| WR_post_, ¬ML (***g = 0***) vs. Bar, ¬ML (***g = 0.6***) | Bar, ¬ML | **5** |
| Bar, ML (***g = 0.5***) vs. Bar, ¬ML (***g = 0***) | Bar, ML | **7** |

**The first column shows the comparison of each pair (A,B) including the hedges’ g values of A winning over B (left) as well as B winning over A (right). The second column shows the winner of each pair. Finally, the third column shows the order in which pairs were locked based on the majority starting with the largest.**

Subsequently, we obtained the ranking of the combinations by locking the results in a directed graph. The adjacency matrix of this graph as well as each pair’s indegree is represented in Appendix S2.3. Edges are directed from row to column. The source of the resulting graph the bar presented interspersed by periods of mean luminance. With an indegree of 1, WR_pre_ presented interspersed with mean luminance periods as well as the bar presented without mean luminance periods were ranked second. WR_pre_ without mean luminance periods was ranked third with an indegree of 2. WR_post_ with mean luminance periods was ranked fourth with an indegree of 3. Finally, WR_post_ without mean luminance periods was ranked fifth with an indegree of 4.

**Appendix S2.3: Directed Graph for pRF Estimate Similarity**

|  | | WR_pre_ | | WR_post_ | | Bar | |
| --- | --- | --- | --- | --- | --- | --- | --- |
|  |  | ML | ¬ML | ML | ¬ML | ML | ¬ML |
| WR_pre_ | ML | **0** | **0** | **1** | **1** | **0** | **0** |
|  | ¬ML | **0** | **0** | **0** | **1** | **0** | **0** |
| WR_post_ | ML | **0** | **0** | **0** | **0** | **0** | **0** |
|  | ¬ML | **0** | **0** | **0** | **0** | **0** | **0** |
| Bar | ML | **1** | **1** | **1** | **1** | **0** | **1** |
|  | ¬ML | **0** | **1** | **1** | **1** | **0** | **0** |
| Indegree |  | **1** | **2** | **3** | **4** | **0** | **1** |

**The graph depicts binary edges leading from row to column as well as the sum total of incoming edges for each vertex (stimulus - mean luminance combination).**

Based on the rankings of the combinations of stimulus type and continuance we derived matrices of voting results for each of them separately. Appendix S2.4 shows the voting results with regard to stimulus.

**Appendix S2.4: Stimulus Voting Results for pRF Estimate Similarity**

|  | WR_pre_ | WR_post_ | Bar |
| --- | --- | --- | --- |
| WR_pre_ |  | **4** | **0** |
| WR_post_ | **0** |  | **0** |
| Bar | **3** | **4** |  |

**Each cell represents the sum of rankings where the row stimulus was ranked above the column stimulus.**

Since the bar was ranked consistently above the other stimuli, it constituted the Condorcet winner for these simulations.

With regard to continuance, Appendix S2.5 shows the voting results for the comparison of presentations with and without interspersed mean luminance periods.

**Appendix S2.5: Mean Luminance Voting Results for pRF Estimate Similarity**

|  | ML | ¬ML |
| --- | --- | --- |
| ML |  | **5** |
| ¬ML | **1** |  |

**Each cell represents the sum of rankings where the continuance (absence or presence of mean luminance periods) given by the row was ranked above the continuance given by the column.**

Since for the majority of rankings the presence of mean luminance periods was preferable to their absence, the inclusion of mean luminance periods constituted the Condorcet winner for the simulations.
